# Recommendations for future university pandemic responses: What the first COVID-19 shutdown taught us

**DOI:** 10.1371/journal.pbio.3000889

**Published:** 2020-08-27

**Authors:** Carolyn Coyne, Jimmy D. Ballard, Ira J. Blader

**Affiliations:** 1 Department of Pediatrics, University of Pittsburgh School of Medicine, Pittsburgh, Pennsylvania, United States of America; 2 Department of Microbiology and Immunology, University of Oklahoma Health Sciences Center, Oklahoma City, Oklahoma, United States of America; 3 Department of Microbiology and Immunology, University at Buffalo, Buffalo, New York, United States of America

## Abstract

The SARS-CoV-2 epidemic challenged universities and other academic institutions to rapidly adapt to urgent and life-threatening situations. It forced most institutions to shut down nearly every aspect of their research and educational enterprises. In doing so, university leaders were thrust into unchartered waters and forced them to make unprecedented decisions. Successes and failures along the way highlighted how the autonomous nature of the American academic research enterprise and skillsets normally required of university leaders were ill-suited to mounting an emergency response. Here, as faculty from medical centers in the United States, we draw lessons from these experiences and apply them as we plan for the next possible COVID-19-induced shutdown as well as other large-scale pandemics and emergencies at universities in the United States and throughout the world.

## Abstract 2

The COVID pandemic brought about unprecedented challenges to universities that altered their teaching and research missions. This Perspective article examines how universities met these challenges and proposes recommendations for university leaders as they meet future, and unfortunately likely, pandemic shutdowns.

## Introduction

The global SARS-CoV-2 pandemic forced universities throughout the world to shift their missions to address this threat. Ultimately, their responses were largely successful and SARS-CoV-2 infections and spread were undoubtedly mitigated during the ensuing shutdowns. However, these responses were not always executed smoothly, in part because of the unparalleled nature of the pandemic. Because we may well face another SARS-CoV-2-associated shutdown, we must consider how effective these responses were. This Perspective’s goal is to generate a framework for these discussions and provide views of faculty members from the proverbial trenches. Although our perspectives are those of medical school faculty, we expect that many of the issues we observed will apply to other academic venues in the United States and throughout the world. In preparing to write this Perspective, we solicited opinions directly from colleagues as well as through social media (e.g. Twitter). Highlights of these communications will be presented below. They are redacted for confidentiality.

### The response

Initial SARS-CoV-2 responses focused on students since education and training are important missions of universities. There were two compelling reasons for this focus. First, the pandemic overlapped with many academic holidays (‘Spring Breaks’) when students leave their campuses, which increased their risk of SARS-CoV-2 exposure that could be spread across campuses upon their return. Second, students in academic health programs (e.g. medicine, dentistry, and nursing) perform clinical rotations, which could expose them and their potentially high-risk patients to SARS-COV-2. Thus, universities quickly and decisively cancelled in-person classes and training. Courses were delivered by video conferencing (e.g. WEBEX, Zoom) with the goal of protecting the institutions while transitioning to a “business as usual” model.

But there were hurdles and barriers. Many faculty members were not familiar with online conferencing software, lacked required tools (e.g. Webcams, high quality microphones), and received limited, if any, training in online content delivery. In addition, significant increases in online content stretched bandwidth capabilities of most institutes. While these issues were expected, others were less so. For example, abrupt dorm closures required students to find alternative housing with high-speed internet access to attend their classes. In addition, students (and faculty) with children or other dependents required homeschooling and alternative care plans that conflicted with classes they either were enrolled in or taught. Thus, socioeconomic disparities undoubtedly affected students’ opportunities and performances. Mental and physical health resources were also impacted, thereby preventing students, faculty, and staff from accessing necessary care and treatment. This was particularly acute given the adverse mental health consequences of the pandemic and subsequent lockdown.

Besides didactic classes, medical school clerkships and laboratory-based classes were also cancelled. Since these are often required for certification and license, cancellations had significant impacts on career progressions. Some schools provided web-based content and/or de-identified case presentations. But, it remains unclear how accreditation agencies will assess these substitutions.

Graduate student courses and presentations also were shifted to online formats. But requirements to work in their laboratories went unchanged. This contradiction laid bare a long-standing issue of how the role of graduate students in the research enterprise is defined–trainees vs. skilled workforce. In many cases, universities allowed individual faculty to determine their laboratory’s status (e.g. open, closed, minimally staffed) as well as to identify “essential” laboratory. In many cases, personnel and trainees were limited in contesting their mentors’ decisions.

Finally, the shutdown posed significant and specific challenges to foreign students. Dorm closures were often executed without housing solutions in place for those requiring alternatives and/or were ineffectively communicated. One option was to return home, but the rapid and changing landscape surrounding travel and visa policies made that option tenuous.

As universities recognized the pandemic’s dangers, they began plans to shut down research laboratories. Some staff moved offsite and focused on project planning, writing, and data analysis. But, the shutdown was not as straightforward for essential duties. For example, laboratory animals require continued care. In many cases, decisions and plans were enacted with key entities collaborating with the overarching goal of maintaining personnel safety. These directives were largely unilateral and delivered to faculty in unambiguous terms. Other swiftly implemented decisions included accommodating research groups who possessed expertise to work on SARS-CoV-2 while creating protocols such as social distancing and PPE use for their safety.

Feedback from social media, conversations with colleagues, and our own observations revealed that decision-making regarding “non-essential” research areas was less efficient and unilateral. There were three primary reasons for this: i) researchers who believed their work was essential despite tenuous relations to SARS-CoV-2; ii) others who had difficulty accepting that their work was not essential; and iii) ambiguous directives from local, state, and federal agencies that were left open to interpretation. For example, the State of Pennsylvania’s edict that Agriculture is an essential industry was interpreted to mean that non COVID-19 related agricultural research was also essential and could continue while non COVID-19 medical research could not [1]. Institutes needed to review petitions and decide which laboratories remained open and often did so under pressure from PIs. In rare cases, investigators ignored shutdown orders and continued laboratory operations as normal. These instances revealed the difficulties universities face in enforcing their own directives.

Clear communication from university leadership was often lacking. For example, one coauthor’s institution remained largely silent until well past SARS-CoV-2 was designated a global pandemic. In addition, messaging from the other co-authors’ universities, and those of colleagues who communicated via social media, was mixed and unclear. Indeed, of 200 respondents to a Twitter poll, >48% indicated that institutional guidance was lacking (https://twitter.com/scienceCC/status/1245016170606088192). Many investigators were confused by which guidelines were being implemented, delaying their ability to communicate directives to their research groups. This led to laboratory staff being required to work without clear mandates and/or mechanisms to be excused. This point is critical, as power dynamics within academic laboratories put staff and trainees in situations where retribution is possible.

Finally, damage to career trajectories was felt at almost every level. Students’ and fellows’ progression towards completing their studies were halted as was faculty career development, which disproportionately affected junior faculty. While most institutes extended tenure clocks, other issues have yet to be resolved and/or addressed including how to recoup funding lost or lapsed during the shutdown.

### Power structures at universities as a barrier to crisis management

Almost every academic health center faced identical circumstances as SARS-CoV-2 spread across the country. University leaders were in the unenvious position of making critical decisions based on rapidly evolving information. While employee and student safety was of paramount concern, compliance with accrediting agencies and integration with hospital partners needed to be considered. In addition, decisions needed to align with local, state, and national government shutdown orders. Compounding this was a lack of testing capabilities that led to uncertainty about infection prevalence.

All of these factors challenged how impactful decisions are made in universities. Effective university leaders are accustomed to making major decisions after input from various stakeholders and through shared-governance. Under routine circumstances this system benefits everyone—faculty are heard and leaders are well aware of a decision’s “buy-in” before its announcement. Academic institutions, by their very nature, do not hire leaders for their ability to make fast and difficult decisions in acute disasters or crises, as these are rare events. Thus, sweeping pronouncements from university leaders are uncommon. In contrast, effective leaders during a crisis are those who can make unilateral decisions, communicate them clearly and unambiguously, and motivate people to execute their plans. In our opinion, the skillsets that make university leaders effective under normal circumstances could very well be an impediment in a time of crisis because many lack the proficiencies and experience to guide their institutions’ responses to catastrophic events.

The shutdown also exposed weaknesses in university leadership structures. These included expansive administrative structures of many institutions, which complicated the process of identifying and mobilizing key leaders. In addition, there were instances of “death by committee”, whereby leaders created committees, which delayed responses by diverting precious time and resources to the task of assembling, organizing, and empowering them. Thus, as this pandemic continues, and other crises arise, universities must reconsider whether their leadership is equipped and has the requisite skillsets to manage their institutions during crises.

### Recommendations

Our aim is to develop a framework for leadership and faculty to assess their responses. We recognize that others may have opposing opinions and/or additional suggestions. Our hope is to initiate discussions so that clear and effective plans are in place before future crises arise. Specific recommendations are discussed below.

1. **Crisis management team preparation, training, and membership**:
  We propose that, if they have not done so already, individuals (e.g. Presidents, Provosts, and Deans) with the ultimate responsibility for making and implementing action plans undergo crisis management training. In addition, rosters for task forces and advisory groups responsible for helping to draft these plans should be in place before a crisis arises. Importantly, membership should be diverse and include those of all academic ranks and include equality of sex and race. During SARS-CoV-2, the gender and race disparities that exist in medical schools were unfortunately reflected in these committees. Committees and working groups should also include mental health experts, so that university leadership can access their expertise as they develop and execute plans. Finally, a single person/entity is needed to coordinate these efforts and who has the centralized authority, resources, and unilateral responsibility to put response plans into action.
2. **Education**:
  Universities need to develop and distribute situational criteria to determine when classes should be moved online. Second, accommodations are required to enable students to participate in delivery of online learning sessions. This includes recording online sessions to enable students to hear lectures missed due to non-academic obligations. In addition, financial consequences of such decisions must be considered and aid provided to students and faculty for purchasing necessary equipment. Third, students must recognize that faculty will likely accrue responsibilities (e.g. homeschooling) and that flexibility is needed in scheduling lectures and meetings. Similarly, departmental and college leaders need to have frank conversations with their faculty and provide necessary resources. Open lines of communication are essential such that faculty can discuss struggles without fear of being viewed negatively.
  Besides didactic lectures, journal clubs, student talks, qualifying exams, thesis committee meetings also began taking place online. While some (e.g. final defenses) are urgent, others are less so. Prioritizing online sessions is important as “burn-out” from online sessions is emerging as a problem [2]. We therefore recommend that priorities be established before the next shutdown so that faculty and students know of and understand expectations.
  Regardless of whether they are freshman, graduate students, postdoctoral fellows, or medical residents, commencing studies is stressful under normal circumstances. To alleviate this stress and anxiety, many schools use programs that promote a sense of community within each cohort group. Thus, the impact of policies such as social distancing and online education on new student acclimation will need to be considered. This could be accomplished by holding regular “virtual happy hours” and town hall-type meetings to facilitate social engagement between incoming and existing students.
3. **Essential versus non-essential research designations**:
  It is critical that universities establish clear procedures and protocols for defining essential research. Clarity would mitigate the adverse impacts on morale that many investigators felt after suspending their non-essential research, only to observe others not doing the same. Ideally, these distinctions would occur while avoiding conflicts of interest (e.g. not allowing faculty to make decisions about their own research). In addition, institutions need to consider the needs of laboratory staff and trainees and include them in the decision-making process.
4. **Human resources/career development**:
  The lessons learned from this shutdown should become part of any institution’s future SOPs. This includes essential personnel designation, telecommuting rules and regulation, and ensuring uninterrupted salary disbursements. It would be critical to engage laboratory staff and trainees in these protocols, particularly in defining who is essential and what mechanisms are in place to appeal these designations.
  Although many schools created contingency plans to provide tenure extensions, they were announced only after junior faculty felt significant angst and concern. Thus, tenure and promotion bylaws should be modified with specific rules that would be automatically implemented in case of future shutdowns. Mechanisms are also needed to support faculty whose funding will lapse or be delayed due to a shutdown. These are obviously difficult decisions since different institutes have different levels of endowments and savings and unique rules for utilizing them. But we believe that institutions are obliged to limit impacts on individual faculty, trainees, and staff. Transparency is also essential in planning how to tackle financial shortcomings.
  Shutdown plans must emphasize mitigating consequences on vulnerable populations. For example, many women with children often became primary caregivers, which undoubtedly will delay career progressions. Institutions (and funding agencies) must address these disparities so that an already leaky pipeline isn’t exacerbated. Finally, those most impacted by these imbalances should be partners in developing these plans and mechanisms created to provide, in real time, feedback so that corrections can be made.
5. **Communication**:
  Institutional leaders must provide updates regarding the status of the shutdown process, even if those updates are merely “we are working on it”. These communications must also be delivered with a single voice, as conflicting messages create tension and confusion. Because of time demands put on leaders during a crisis, we recommend creating a crisis communications team that is directed by and answers to the highest academic officer (e.g. university president or provost). Faculty, staff, and trainees should be encouraged to ask questions, even if it means going outside of the “chain of command”. Finally, institutions should appoint pandemic ombudsmen to confidentially address individual complaints and concerns.

## Conclusions and perspectives

More pandemic-related shutdowns likely lie ahead and institutions will face the prospect of making the same decisions. We hope that as university leaders and faculty review their actions and decisions during this first shutdown that they will not only focus on successes but also identify and learn form their deficiencies.
